# Efficacy of fixed-dose combination artesunate-amodiaquine *versus* artemether-lumefantrine for uncomplicated childhood *Plasmodium falciparum* malaria in Democratic Republic of Congo: a randomized non-inferiority trial

**DOI:** 10.1186/1475-2875-11-174

**Published:** 2012-05-25

**Authors:** Emmanuelle Espié, Angeles Lima, Benjamin Atua, Mehul Dhorda, Laurence Flévaud, Eric M Sompwe, Pedro Pablo Palma Urrutia, Philippe J Guerin

**Affiliations:** 1 Epicentre, 8, rue Saint Sabin, 75011, Paris, France; 2 Médecins Sans frontières - Operational Centre Barcelona-Athens, Barcelona, Spain; 3 National Malaria Programme, Ministry of Health, Kinshasa, Democratic Republic of Congo; 4 Epicentre research base, Mbarara, Uganda; 5 Ministry of Health, Katanga Province, Lubumbashi, Democratic Republic of Congo; 6 Centre for Tropical Medicine, Nuffield Department of Clinical Medicine, University of Oxford, Oxford, UK

**Keywords:** Uncomplicated malaria, Artesunate-amodiaquine, Artemether-lumefantrine, Child, Treatment efficacy

## Abstract

**Background:**

In 2005, the Democratic Republic of Congo (DRC) adopted artesunate and amodiaquine (ASAQ) as first-line anti-malarial treatment. In order to compare the efficacy of the fixed-dose formulation ASAQ *versus* artemether-lumefantrine (AL), a randomized, non-inferiority open-label trial was conducted in Katanga.

**Methods:**

Children aged six and 59 months with uncomplicated *Plasmodium falciparum* malaria were enrolled and randomly allocated into one of the two regimens. The risk of recurrent parasitaemia by day 42, both unadjusted and adjusted by PCR genotyping to distinguish recrudescence from new infection, was analysed.

**Results:**

Between April 2008 and March 2009, 301 children were included: 156 with ASAQ and 145 with AL. No early treatment failures were reported. Among the 256 patients followed-up at day 42, 32 patients developed late clinical or parasitological failure (9.9% (13/131) in the ASAQ group and 15.2% (19/125) in the AL group). After PCR correction, cure rates were 98.3% (95%CI, 94.1-99.8) in the ASAQ group and 99.1% (95%CI, 94.9-99.9) in the AL group (difference −0.7%, one sided 95% CI −3.1). Kaplan-Meier PCR-adjusted cure rates were similar. Both treatment regimens were generally well tolerated.

**Conclusion:**

Both ASAQ and AL are highly effective and currently adequate as the first-line treatment of uncomplicated *falciparum* malaria in this area of Katanga, DRC. However, in a very large country, such as DRC, and because of possible emergence of resistance from other endemic regions, surveillance of efficacy of artemisinin-based combination treatments, including other evaluations of the resistance of ASAQ, need to be done in other provinces.

**Trial registration:**

The protocol was registered with the clinicaltrials.gov, open clinical trial registry under the identifier number NCT01567423.

## Background

Despite recent progress in access to effective preventive and therapeutic measures to control malaria, it remains one of the leading causes of morbidity and mortality worldwide, and especially in Democratic Republic of Congo (DRC) [1]. In this country, malaria cases was reported to represent up to 68% of outpatient visits and 30% of hospital admissions and accounted for an estimated 42% mortality in children under five, all over the country [2]. Malaria is seasonal, with transmission peaking during the rainy season from September to May and *Plasmodium falciparum* accounts for nearly 95% of all malaria cases in this region [3].

In 2005, following increased reports of sulphadoxine-pyrimethamine (SP) resistance [4], the National Malaria Control Programme the Ministry of Health of DRC decided to replace SP by artesunate (AS) combined with amodiaquine (AQ), as first-line treatment, following WHO guidelines recommendation to use artemisinin-based combination therapy (ACT) for uncomplicated malaria [5]. The association was initially only available in loose combination of ASAQ in blister pack.

Until 2007, the combination of artemether-lumefantrine (AL) was the only fixed-dose ACT registered to international standards that was widely available in malaria-endemic countries. A new fixed-dose combination of artesunate and amodiaquine (ASAQ Winthrop®), developed by the Drugs for Neglected Diseases Initiative (DNDi) in collaboration with Sanofi-Aventis, was pre-qualified by the WHO in 2008.

Current international guidelines advocate that once a new anti-malarial treatment is introduced in a country, the efficacy of such novel regimens needs to be monitored regularly in order to detect early signs of declining efficacy, which can have implications for policy markers [5]. The few studies conducted in eastern DRC showed that the efficacy of ASAQ by day 28 varied between 84.9% and 93.3% in children less than 5 years old [4,6,7]. As DRC is a very large country of approximately 2,345,000 km^2^, repeated evaluations of the ACT efficacy are needed in different regions.

Given that the transition in drug policy from a loose to a fixed dose combination would be easier option for uncomplicated malaria treatment, it was decided to assess the efficacy of the new fixed-dose combination of artesunate and amodiaquine (ASAQ) in comparison with the fixed-dose combination of artemeter and lumefantrine (AL), in the province of Katanga, south-eastern DRC.

## Methods

The aims of the study were to compare (i) treatment efficacy of fixed-dose ASAQ and its alternative AL, expressed as product limit estimates of failure from survival analysis and as simple proportions from per protocol analysis, on day 42, and (ii) analysis of adjusted and unadjusted results by genotyping.

### Study design and site

**Figure 1:**
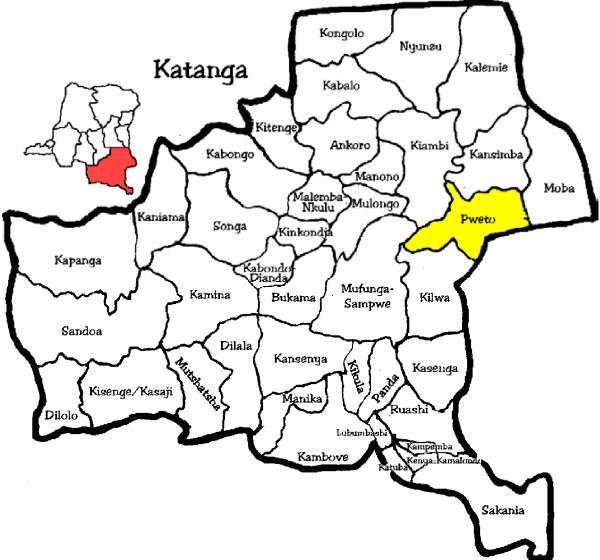
Map of Democratic Republic of Congo.

An open randomized study was conducted to test the hypothesis that the risk of recurrent parasitaemia after 42 days is not inferior in the group receiving ASAQ regimen compared to the group receiving AL regimen. The patients were recruited from the outpatient department of the general reference hospital of Pweto, health district of Pweto, province of Katanga, DRC, between April 2008 and March 2009 (Figure 1).

This protocol was registered with ClinicalTrials.gov, under the idenfier number NCT01567423.

### Procedures

Children aged between six and 59 months and with a body weight ≥ 5 kg were eligible for enrolment if they had *P. falciparum* infections (density threshold at inclusion between 2,000 and 200,000/μl), fever or history of fever in the previous 24 hours, no signs of severe malaria, no reported hypersensitivities of the studied drugs, and no serious concomitant febrile illness [5].

The children included were randomized in one of the two treatment arms, in a 1:1 ratio without stratification. The randomization list was generated by a computer in blocks of six. Treatment allocations were kept in sealed and numbered opaque envelopes. Participants were enrolled in the same order in which they were diagnosed.

ASAQ (ASAQ Winthrop®, Sanofi-Aventis) was administered once daily for three days, as follows: one tablet of artesunate 25 mg/amodiaquine 67.5 mg for children between 5 to 8.9 kg, artesunate 50 mg/amodiaquine 135 mg for children between 9 to 17.9 kg. One tablet of artemether 20 mg/lumefantrine 120 mg (Coartem®, Novartis Pharma, Basel, Switzerland) was administered twice daily for three days to children with a body weight of 5 to 14.9 kg and two tablets were administered twice daily for three days to children with a body weight of 15 to 24.9 kg. All doses were administered under direct observation for the three days. Full doses of drugs were re-administered if a patient vomited within 30 min after receipt. Patients who vomited more than twice were excluded from the study and were treated with oral quinine 10 mg/kg/8 h for 7 days.

Signs of severe malaria or any other serious health condition (*e.g.* severe malnutrition), intake of anti-malarial treatment in the last seven days, and mixed malaria infection were also excluded from the study.

Clinical assessment (including measurement of axillary temperature), tick and thin smears and haemoglobin measurement were performed on days 2, 3, 7, 14, 21, 28, 35 and 42 or any day in between in the event of illness.

### Malaria outcome classification

Outcomes were classified according to 2009 WHO guidelines as adequate clinical and parasitological response (ACPR), early treatment failure (ETF), late clinical failure (LCF), late parasitological failure (LPF) or follow-up interrupted [8]. Follow-up interrupted included treatment protocol violation, lost to follow-up, use of other anti-malarials outside the study protocol or withdrawal of consent prohibiting further follow-up. Drug tolerability was also assessed clinically. An adverse event was defined as any undesirable symptom in a patient during the study regardless of whether it was related to the treatment.

### PCR genotyping

In areas of intense transmission, where multiple genotype infections are common [9], a second episode of malaria or a recurrent parasitaemia during the drug-free follow-up period may be due to the same infection or a different infection (a recrudescence, thus a treatment failure, or a new infection, respectively). To distinguish these two events, polymorphic *P. falciparum* genes, such as the merozoite surface protein 1 and 2 genes (*msp1* and *msp2*) and the glutamate-rich protein gene (*glurp*) were genotyped by PCR, as previously described [10]. PCR analyses, conducted at the Epicentre research base, Mbarara, Uganda, were performed using paired samples from all patients experiencing late clinical or parasitologic failure. Blood samples were collected on Whatman FTA® Cards (GE Healthcare UK Limited, Buckinghamshire, United Kingdom) on the day of enrollment and the day treatment failure occurred. The genotypic profiles for pre- and post-treatment parasites were compared; patients in which pre- and post-treatment genotypes were identical were considered as recrudescences and patients in which pre- and post-treatment genotypes were different were considered as new infections [11].

All ETFs were considered to be due to recrudescence. Patients meeting the criteria for LCF or LPF in whom genotyping was done; according to the results, patients were classified as either (i) resolved by PCR and further categorized as recrudescences or new infections or (ii) unsuccessfully genotyped with the reason recorded (missing sample, PCR not done or result inconclusive).

### Ethics

This study underwent ethical review and was approved by Ethical Committee of the School of Public Health, University of Kinshasa, DRC, the ethical review board of Médecins Sans Frontières and the Comité de Protection des Personnes “Ile de France XI”, St Germain en Laye, France. The study was authorized by the relevant health authority. Written informed consent was obtained from the parents or legal guardians of the enrolled children.

### Statistical analyses

Based on previous data for AL efficacy [12-14], efficacy rate, defined as the PCR-adjusted parasitological cure rate at day 42, was estimated of 95% for both treatments. With a 7% non-inferiority margin, a power of 80%, and a test significant level (one side) of 5%, the sample size required to conclude to non inferiority was 150 per treatment group, taking into account an increase of 20% for loss to follow-up or premature withdrawals [nQuery Advisor v6].

The primary outcome was the PCR-adjusted parasitological cure rate up to day 42 of the follow-up period. Two analytical approaches were used to assess efficacy data. First, a per protocol (PP) analysis was performed including only the patients who were followed throughout the protocol, defined follow-up period and in whom a clear treatment outcome can be determined. The risk of failure for each treatment group was calculated as the proportion of patients classified as failure (the numerator) divided by the number of patients in the evaluable population (the denominator). In the second approach, survival analysis was performed and patients with incomplete follow-up who did not reach the primary outcome interest were included in the analysis as non-failures, but censored on the last day of follow-up. The risk of failure was calculated using the Kaplan-Meier product limit formula with data censored for patients who were not classified as failures and with interrupted follow-up. Patients wrongly included, who did not meet study inclusion criteria, were excluded from both analyses.

In the PP analysis (adjusting by genotyping), the evaluable population included only patients classified as ACPR, ETF or LCF/LPF due to recrudescence. In the survival analysis, the evaluable population for adjusted and unadjusted calculations included all patients enrolled in the study, with the exception that LCF/LPF outcomes with unsuccessful genotyping outcomes were excluded from the adjusted calculations. For the unadjusted calculations, patients with follow-up interrupted and non-*falciparum* new infections were censored on the last day of observation. For the adjusted calculations, censored patients also included those with new *P. falciparum* infections.

Other variables were compared using the chi2 test or Fisher’s exact test for variables and Student’s test for continuous variables.

Data were double entered and validated using Epidata version 3.1 (Odense, Danemark). All analyses were performed with Stata, version 10 (Stats-Corp, College Station, Texas). A p-value < 0.05 was considered statistically significant.

## Results

Between April 2008 and March 2009, a total of 1,993 patients were screened, 301 children aged between six and 59 months were enrolled in the two treatment arms, 156 with ASAQ and 145 with AL. The mean age of patients at baseline was 27.5 months (range 6–59 months) and 152 (50.5%) were females. There were no differences between treatment arms at study inclusion, for all variables studied, including demographic, clinical and laboratory characteristics, confirming adequate randomization (Table 1).

**Table 1:** Baseline characteristics of trial participants, by treatment group

	**ASAQ**	**AL**	
Patients included	156	145	-
Age (months)	27.6 (6–59; 14.3)	27.4 (6–59; 15.0)	
Sex (female)	83 (53.3%)	69 (47.6%)	
Weight (kg)	10.3 (5.1-17.0; 2.7)	10.2 (5.3-16.0; 2.6)	
Axillary temperature (°C)	37.6 (34–40.5; 1.3)	37.6 (35.2-40.0; 1.1)	
Patients with fever (T° ≥ 37.5°C)	79 (50.6%)	71 (48.9%)	
Haemoglobinaemia (g/dL)	9.0 (4.0-13.3; 1.9)	9.0 (5.3-15.5; 2.1)	
Anaemia 5- < 8 g/dl	50 (32.0%)	51 (35.2%)	
8-11 g/dl	83 (53.2%)	65 (44.8%)	
> 11 g/dl	23 (14.8%)	29 (20.0%)	-
Geometric mean parasite density (/μL) (IQR)	17111 (34674)	14886 (53420)	
Proportion ≥ 100,000 para/μL	13 (8.3%)	11 (7.5%)	
Patients with gametocytes	9 (5.8%)	7 (4.8%)	

Data are number (%) or mean (range; SD), unless otherwise indicated.

**Figure 2:**
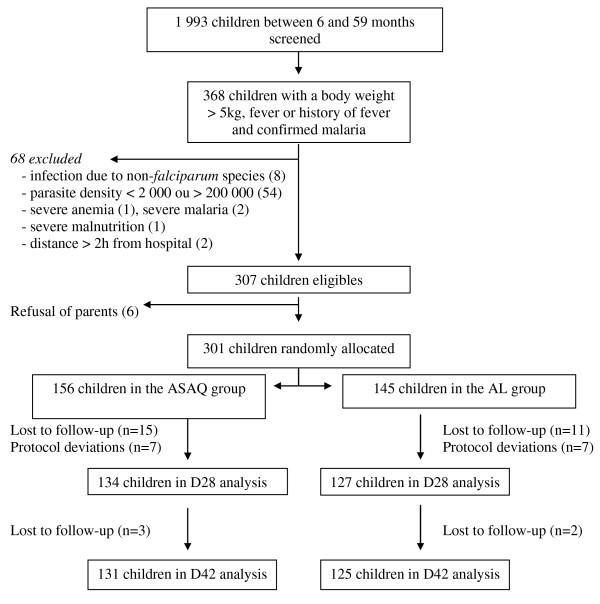
**Trial profile.** ASAQ, artesunate-amodiaquine; AL, artemeter-lumefantrine.

The clinical trial profile is presented in Figure 2. Thirty-one (10.3%) were lost during follow-up (26 before day 28, five on or after day 28); and 14 (4.6%) were withdrawn (all before day 14). The reasons for withdrawal were co-infection with *P. falciparum* and *Plasmodium malariae* at study inclusion (8/14), taking another anti-malarial drug (1/14), severe anaemia (1/14) and other violation protocol (4/14). All these 14 patients were excluded from the analyses.

### Adequate clinical and parasitological response and treatment cure rates

Among the 256 patients still followed-up at day 42, adequate clinical and parasitological response was observed in patients: 90.1% (118/131) in the ASAQ group and 84.8% (106/125) in the AL group. Overall, no patient experienced early treatment failure, and 32 patients developed late clinical or parasitological failure (9.9% (13/131) in the ASAQ group and 15.2% (19/125) in the AL group) (Table 2).

**Table 2:** Treatment outcomes after 42 days for episodes of uncomplicated *Plasmodium falciparum* malaria

**Treatment outcome**	**Treatment group, No. (%)**	
	**ASAQ (n = 156)**	**AL (n = 145)**
No treatment outcome	25 (16.0)	20 (13.8)
Lost to follow-up	18	13
Other protocol deviations	7	7
Adequate clinical and parasitological response	118 (75.6)	106 (73.1)
Recurrent parasitaemia (including LCF and LPF)	13 (7.4)	19 (13.1)
Recrudescence	2	1
New infection	9	17
Recurrent malaria due to non-*falciparum* species	1	1
Genotyping not performed	1	0

Among the 32 children with recurrent parasitaemia after day 7, PCR genotyping was successfully performed on 31 (96.8%) of 32 blood samples (Table 2). PCR-corrected cure rates by day 42 were 98.3% (95%CI, 94.1-99.8) following completion of the ASAQ regimen and 99.1% (95%CI, 94.9-99.9) following completion of the AL regimen (difference −0.7%, one sided 95% CI −3.1).

**Figure 3:**
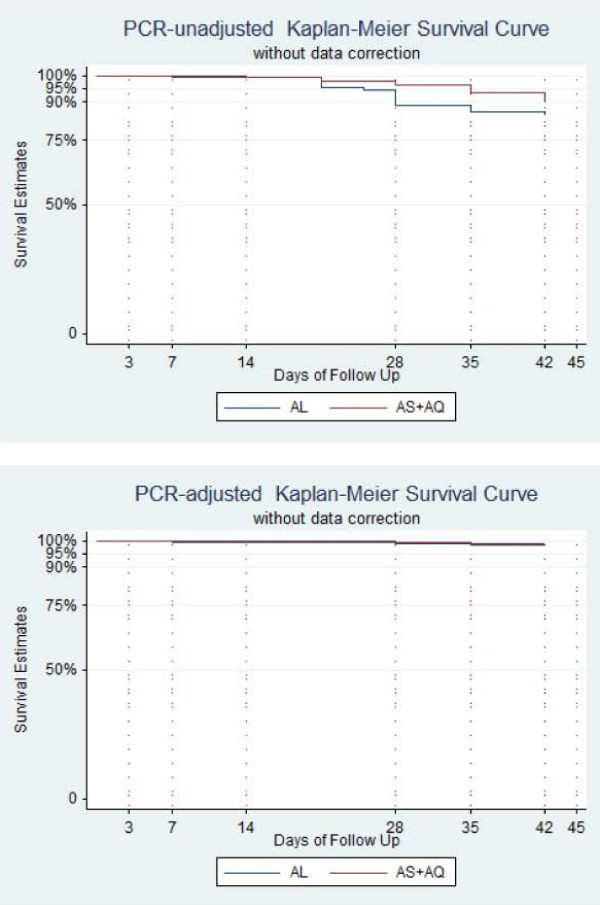
Kaplan Meier curves, a) PCR-unadjusted b) PCR-adjusted. ASAQ, artesunate-amodiaquine; AL, artemeter-lumefantrine.

Table 3 and Figure 3 gives the results of unadjusted and PCR-adjusted Kaplan Meier cure rate analysis at day 42.

**Table 3:** Efficacy outcome for ASAQ and AL on day 42, calculated by PP analysis and Kaplan-Meier analysis

	**PP cure rate analysis**		**Kaplan Meier cure rate analysis**	
	**ASAQ**	**AL**	**ASAQ**	**AL**
PCR unadjusted				
n/N	13/131	19/125	12/156	18/145
% efficacy [CI95%]	90.1 [83.6-94.6]	84.8 [77.3-90.6]	90.9 [84.6-94.8]	85.8 [78.4-90.8]
% difference (LLCI*)	−5.2 (−0.02)			
PCR adjusted				
n/N	2/120	1/107	2/146	1/137
% efficacy [CI95%]	98.3 [94.1-99.8]	99.1 [94.9-99.9]	98.4 [93.8-99.6]	99.2 [94.3-99.9]
% difference (LLCI*)	−0.7 (−3.1)			

*LLCI : lower limit of the one-sided 90%CI.

### Parasite and fever clearance, and gametocyte carriage

Clearance of fever (defined as a temperature <37.5°C) was more rapid in patients given ASAQ than in patients given AL, one day after treatment initiation. Among patient who had fever at inclusion, one (1.3%) of 79 ASAQ recipients and eight (11.3%) of 71 AL recipients remained febrile (p-value = 0.002). By day 2, nearly all fevers had resolved and the two regimens did not differ (p-value = 0.38). Rapid clinical improvement was recorded within the two treatment groups.

By day 2, parasite clearance did not differ between the two regimens, with positive slide results observed for nine (6.0%) of 150 ASAQ-treated patients and for seven (4.9%) or the 143 AL-treated patients (p-value = 0.68). By day 3 and day 7, only one patient (respectively in the AL group and in the ASAQ group) had not yet cleared all parasites.

At baseline, gametocyte carriage rates were low in both regimen groups (Table 1). Gametocytes were detected more often with ASAQ than with AL, respectively by day 2, 11.9% and 4.8%, by day 3, 10.6% and 4.2%, and by day 7, 6.3% and 1.5% (p-value < 0.05).

### Adverse events

Both treatment regimens were generally well tolerated. Twelve patients (ten (6.4%) in the ASAQ group and two (1.4%) in the AL group) vomited within 30 min after the administration of the dose; nine of them (all in the ASAQ group) had received a second dose and none was excluded from the study.

No deaths occurred during the follow-up period, but six patients (one in the ASAQ group and five in the AL group) developed moderate or severe adverse events associated with clinically suspected severe malaria (anaemia, lethargy, convulsions). All patients were transfused and one received the rescue treatment consisting on intravenous quinine (loading dose 20 mg/kg given over 4 hours, then 10 mg/kg given 8 hours after the loading dose was started, followed by 10 mg/kg every 8 hours for 7 days). Four of these six patients (all in the AL group) were hospitalized for two to six days. Thus, these events could not be attributed to the intervention drugs.

Other mild side-effects were less common and were not related to a specific treatment: lower acute respiratory infections (54.6% in the ASAQ group and 51.3% in the AL group), conjunctivitis (16% in the ASAQ group and 10.8% in the AL group), abdominal pains and diarrhoea (8% in the ASAQ group and 14.9% in the AL group).

## Discussion

The results of this randomized trial of Congolese children in Katanga province with uncomplicated *falciparum* malaria and follow-up patients for 42 days show that the efficacy of fixed-dose combination of ASAQ is non-inferior to fixed-dose combination of AL, with regard to the margin prespecified at 7%.

High PCR adjusted cure rates of 98.3% to 98.4% (depending on the analysis population) were seen in patients assigned to ASAQ, compared with rates of 99.1% to 99.2% in patients assigned to AL. These results are comparable to those from previous studies where AS+AQ were routinely used as multiple tablets (loose combination) in other sub-Saharan countries [12-14], but higher than those reported by Bonnet *et al.* in Equatorial province of DRC [7]. They are also consistant with those from recent studies which studied efficacy of fixed-dose combination of ASAQ after 28 days of follow-up [15,16].

As with other malaria efficacy studies conducted in areas of high transmission [13], the unadjusted efficacy rates, which were significantly different between the two treatment arms, largely reflect a difference in rates of new infection than in rates of recrudescence. Thus, in this study, a significant proportion of new infections was reported, most prominently in the AL group, which reflect partially the prophylactic effect of the drugs.

In this study, the adjusted estimates of treatment efficacy derived from the two statistical approaches were not significantly different. New infection with *P. falciparum* usually constitutes an additional confounding factor for the adjusted analysis. Whereas patients with such infections are removed from the PP analyses, they are censored in the survival analysis after contributing a period of observation to the cumulative risk during which treatment failure was not observed [17]. This discrepancy was particularly apparent in the high transmission sites in Africa where new infections were highest, but it was not observed in this study.

Survival analysis allows for more available data to contribute to the analysis, thus increasing the precision of the derived estimates. It avoids systematic biases introduced by dropping from the analysis patients who do not complete follow-up [8,18,19], even if in this study, the proportion of these patients was 10.3%, which could be considered as acceptable.

During the first days of treatment, a rapid decrease of fever, parasitaemia and gametocyte carriage was observed in the two regimen groups. These findings confirmed the results of efficacy studies on artemisinin-containing combination which led to lower gametocyte carriage, improving the cure rates, decreasing the transmission of falciparum malaria and reducing the spread of resistance to non-artemisinin drugs [20,21]. Parasite clearance has became an important indicator in the evaluation of ACT, especially following the emergence of artemisinin resistance in Cambodia and the Thai-Burmese border [22-24]. Here the proportion of patients with low parasitaemia at day 2 and 3 is giving new important indication of the parasite early response following ACT [25].

This study showed that both combinations were well tolerated, with only 6% of children with drug-induced vomiting. Nevertheless, differents between treatment arms were observed in the first few days following treatment; vomiting was higher in the ASAQ group compared to AL. Only four patients developed severe malaria, and no deaths occurred during the course of the study. It is worth mentioning that the study was not designed nor power to compare tolerabilty between the two combinations. The ASAQ FDC presents the advantage of requiring one intake per day while AL is twice per day [26]. However, the tolerability, in particular vomiting may impact the effectiveness of the treatment and should be assessed, particularly while used in home management of malaria [27] or as intermittent preventive therapy in children [28].

A limitation of this study was that pharmacology measures were not performed. It would improve the differentiation between true recrudescence to a problem of absorption [29]. The limited reliability of the electric supply and the absence at that time of standardized PK measure on filter paper were among the reasons why these measures were not implemented.

According to WHO guidelines, which recommend a change in anti-malarial treatment policy when the cure rate for the current recommended therapy falls below 90% [5], this study showed that both ASAQ and AL are currently adequate as the first-line treatment of uncomplicated falciparum malaria in this area of the Katanga province. Surveillance of ACT efficacy in a very large country such as DRC remains a technical, financial and logistic challenge, illustrated by the limited number of published and unpublished efficacy studies in the past decade [4,6,7,30].

While emergence of artemisinin resistance in South East Asia is of serious concern [22,23], possible emergence of resistance from other endemic regions is triggering renewed attention in monitoring anti-malarial efficacy. More financial resources are now available in particular from Global Funds, World Bank or PMI to support this surveillance. However, the current recommended protocol for monitoring anti-malarial efficacy can be very challenging to implement, in particular, in remote study sites. In this particular study, the difficulties to supply equipment, drugs, consumables, establishing external quality assurance processes and accessing the site were among daily challenges. The follow-up of patients in particular in a border area was also a major issue for the study team. Research is needed to develop simpler methods to monitor efficacy, *e.g.*, shorter follow-up of patients, and validate molecular marker of artemisinin resistance.

Initiatives, including development of paediatric formulations, home-based management of malaria and improving public sector procurement and supply chains should make ACT more accessible for sub-Saharan African children [31,32].

## Competing interests

The authors declare that they have no competing interests.

## Authors’ contributions

The authors accept full responsibility for the overall content of this report. EE was the principal investigator of the study. She designed and coordinated the study, managed and analysed the data, and wrote the manuscript. AL participated to the development of the study protocol, gave a technical support to the investigator and revised the manuscript. BA, as director of the National Malaria Control Programme in DRC, gave support and approval of the study and revised the manuscript. LF participated in the development of the study protocol, gave a technical support for the laboratory issues, coordinated the external quality control of the reading of malaria slides and revised the manuscript. EMS, as the local authority and health staff, gave support and approval of the study and revised the manuscript. MD performed the PCR analyses and gave advice on PCR results interpretation. PPPU participated in the development of the study protocol, gave technical support to the investigators and revised the manuscript. JPG gave technical support to the principal investigator during the protocol writing, analysis and writing of the manuscript.
